# Activating Brown Adipose Tissue for Weight Loss and Lowering of Blood Glucose Levels: A MicroPET Study Using Obese and Diabetic Model Mice

**DOI:** 10.1371/journal.pone.0113742

**Published:** 2014-12-02

**Authors:** Chenxi Wu, Wuying Cheng, Yi Sun, Yonghong Dang, Fengying Gong, Huijuan Zhu, Naishi Li, Fang Li, Zhaohui Zhu

**Affiliations:** 1 Department of Nuclear Medicine, Peking Union Medical College Hospital, Chinese Academy of Medical Sciences and Peking Union Medical College, Beijing 100730, China; 2 Department of Endocrinology, Peking Union Medical College Hospital, Chinese Academy of Medical Sciences and Peking Union Medical College, Beijing 100730, China; INSERM, France

## Abstract

**Purpose:**

This study aims at using ^18^F-FDG microPET to monitor the brown adipose tissue (BAT) glucose metabolism in obese and diabetic mouse models under different interventions, and study the therapeutic potential of BAT activation for weight loss and lowering of blood glucose in these models.

**Methods:**

Obese mice were established by a high-fat diet for eight weeks, and diabetes mellitus(DM) models were induced with Streptozocin in obese mice. ^18^F-FDG microPET was used to monitor BAT function during obese and DM modeling, and also after BRL37344 (a β_3_-adrenergic receptor agonist) or levothyroxine treatment. The BAT function was correlated with the body weight and blood glucose levels.

**Results:**

Compared with the controls, the obese mice and DM mice showed successively lower ^18^F-FDG uptake in the interscapular BAT (*P* = 0.036 and <0.001, respectively). After two-week BRL37344 treatment, the BAT uptake was significantly elevated in both obese mice (*P* = 0.010) and DM mice (*P* = 0.004), accompanied with significantly decreased blood glucose levels (*P* = 0.023 and 0.036, respectively). The BAT uptake was negatively correlated with the blood glucose levels in both obese mice (r = −0.71, *P* = 0.003) and DM mice (r = −0.74, *P* = 0.010). BRL37344 treatment also caused significant weight loss in the obese mice (*P* = 0.001). Levothyroxine treatment increased the BAT uptake in the control mice (*P* = 0.025) and obese mice (*P* = 0.013), but not in the DM mice (*P* = 0.45).

**Conclusion:**

The inhibited BAT function in obese and DM mice can be re-activated by β_3_-adrenergic receptor agonist or thyroid hormone, and effective BAT activation may lead to weight loss and blood glucose lowering. Activating BAT can provide a new treatment strategy for obesity and DM.

## Introduction

Active brown adipose tissue (BAT) has been found in adult humans, which is believed to play an important role in metabolic balance in the body [1], [2]. As demonstrated by ^18^F-FDG PET/CT, BAT is usually distributed symmetrically at the cervical-supraclavicular, paravertebral, and periadrenal regions. In some special circumstances, such as in patients with malignant pheochromocytoma [3], the BAT tissues in omental and mesenteric regions can also be significantly activated by cold exposure [4]. When the BATs are activated, the catecholamine released from sympathetic nerve endings will trigger the β_3_-adrenergic receptors and lead to high-level expression of uncoupling protein-1 (UCP1) on the inner membrane of mitochondria, which can burn glucose and fatty acids to produce heat through a process known as non-shivering thermogenesis [5]. Recently, BAT has been emerging as a promising target to treat obesity and diabetes mellitus (DM) through this special energy expenditure pathway [6]–[8], specifically by means of consuming fat in obese patients and lowering glucose level in DM patients [9]. Some human studies have suggested that obesity might be associated with a decreased volume of BAT [10], and mice studies have also shown that absence of BAT activity could aggravate obesity [11], [12]. Blood glucose level has been found to be interrelated with BAT activity, and the “detectable” BAT on ^18^F-FDG PET may serve as a protective factor against DM [13]. Although several related pre-clinical studies have been reported [8], [14], [15], most of these studies are based on *in vitro* biochemical analysis or pathological analysis on BAT, and themetabolic changes of BAT were not monitored *in vivo*. Therefore, the dynamic relationship among BAT metabolism, obesity and DM *in vivo* still warrants further investigation [16].

In our prior study, we tested different interventions in stimulation or inhibition of the glucose metabolism in mice BAT using ^18^F-FDG microPET, and have established a standardized method to evaluate BAT function [17].In this presented study with the goal of investigating the role of BAT in disease progression and its therapeutic value, we monitored BAT function in obese and DM model mice using ^18^F-FDG microPET. We also evaluated BAT function change under various interventions in model mice, including BRL37344 (a specific β_3_-adrenergic receptor agonist) and levothyroxine (a thyroid hormone), both of which own the ability of activating the BAT metabolism [18], [19]. With these observations, we examined the feasibility of ^18^F-FDG microPET imaging of BAT in DM and obesity disease models, and discovered a significant relationship between BAT function, blood glucose level and body weight change. These results suggested ^18^F-FDG PET can provide a broad research platform for potential therapy investigations for DM and obesity, and also, increasing BAT metabolism can be a possible therapeutic method for DM and obesity.

## Materials and Methods

### Animal modeling

All animal experiments were performed under the approval of the Committee of Animal Experimentation of Peking Union Medical College Hospital (PUMCH). The mice were raised in a specific pathogen-free area with a constant room temperature of 20–21°C and a standardized light/dark cycle. All mice had *ad libitum* access to food and water. Five-week-old male Imprinting Control Region (ICR) mice were used in this study. Control mice were fed on basic rodent feed (barley flour 20%, soybean powder 20%, wheat bran 16%, corn flour 16%, vegetables powder 10%, fish meal 10%, bone powder 5%, salt 2%, and yeast powder 1%). Obese mice models were established by feeding a high-fat diet (70% of basic rodent feed, whole milk powder 10%, fat 10%, egg yolk powder 10%, and 10 drops of fish liver oil per kilogram diet, provided by Fukang Biotech. Co., Beijing, China) for eight weeks, and the mice with weight 20% higher than normal controls were selected as obese mice. DM model was induced on the obese mice by injecting Streptozocin (Sigma Aldrich Inc., St. Louis, MO, USA) at 160 mg/kg for consecutive three days. Mice with a fasting blood glucose level >11.1 mmol/L after Streptozocin interventions were selected as DM models.

### Interventions

To activate the BAT, eight obese mice, six DM mice, and six control mice received intraperitoneal injection of BRL37344 (4-[-[[2-hydroxy-[3-chlorophenyl] ethyl]-amino] propyl] phenoxyacetate, Sigma Aldrich Inc., St. Louis, MO, USA) at 2.5 mg/kg three times per week for two weeks, whereas six obese mice, five DM mice, and six control mice received levothyroxine (Euthyrox, Merck KGaA, Germany) intervention at 30 µg/kg via intragastric administration three times per week for two weeks (**Table 1**). A total of nine normal mice that received no treatment were served as controls. The obese and DM mice were maintained on the high-fat diet during the whole experiment.

**Table 1:** Groups of mice used in the studies.

Groups	Mouse number
***Obesity model***	
BRL37344 treatment	8
Levothyroxine treatment	6
Saline	8
***Diabetes mellitus model: BRL37344 study***	
BRL37344 treatment	6
Saline	5
***Diabetes mellitus model: thyroxine study***	
Levothyroxine treatment	5
Saline	6
***Normal mice***	
Single BRL37344 treatment	*6*
Levothyroxine treatment	*6*
Saline	*9*

### ^18^F-FDG microPET imaging and analysis

^18^F-FDG was synthesized automatically using an FDG-N module (PET Co., Beijing, China) according to clinical requirements. The synthesis procedure had been approved by the Radiopharmaceutical Administration and complied with good-manufacturing-practice guidelines. ^18^F-FDG was pyrogen free and was of sufficient quality for clinical use, with a radiochemical purity of >98%.

A Siemens *Inveon* microPET imaging system was used for PET scanning of mice. A 5-min static PET acquisition was performed 1 hour after ^18^F-FDG injection for each mouse. For mice that received the BRL37344 intervention, PET imaging was performed three times during the study at baseline, after establishment of obesity/DM model, and after BRL37344 treatment. For mice that underwent the levothyroxine intervention, a PET scan was performed after the treatment. All mice were fasted overnight before PET scanning. On the day of each PET study, all mice were pre-exposed to cold stimulation by stepping on ice (wrapped with plastic gloves and covered by utility wipes) for 1 h at room temperature before ^18^F-FDG injection. ^18^F-FDG (7.4 MBq) was injected intraperitoneally 1 h before PET scanning. Mice were under isoflurane anesthesia throughout the PET acquisition procedure. The image data were reconstructed using a Filtered Back Projection (FBP) algorithm. Semi-quantitative analysis was performed using the Siemens *Inveon Research Workplace* software. The maximum uptake value (nCi/cc) of ^18^F-FDG in the interscapular BAT and the mean uptake value in the liver were measured by volume-of-interest (VOI) method. The ^18^F-FDG uptake ratio of BAT to liver (BAT/L) was then calculated and compared between the groups.

### *Ex vivo* biodistribution analysis

Biodistribution studies were performed in 5-week-old male ICR mice (n = 12), to observe the distribution of ^18^F-FDG in BAT and some organs with or without interventions. Six mice received an intraperitoneal injection of BRL37344 (2.5 mg/kg) 1 h before injection of 3.7 MBq (100 µCi) ^18^F-FDG, whereas the six control mice received saline 1 h before ^18^F-FDG tracer injection. The mice were kept at room temperature after intervention and tracer injection. Immediately after the PET imaging, all mice were sacrificed and the interscapular BAT, liver, brain, muscle, and mesenteric white adipose tissue (mWAT) were isolated and weighed. Tissue radioactivity was counted using a gamma counter. The radioactive counts per min per gram tissue (CPM/g) were measured and compared between the two groups.

### Blood tests

Blood tests were performed in treated mice and controls at the end of the study. All mice were fasted overnight before blood collection. For each mouse, 150–200 µL blood was collected from the retrobulbar venous plexus using a heparin pre-soaked capillary glass tube. Mice were under anesthesia for the whole blood collection process. The serum was separated through centrifugation (3500 rpm for 10 min at room temperature) and tested for blood glucose (Glu), total cholesterol (TC), and low-density lipoprotein-cholesterol (LDL-C) in the Laboratory Department in PUMCH.

### Body weight monitoring

Body weight of the obese mice was monitored at baseline, after induction of obesity, and after BRL37344 treatment. Body weight of the obese mice after levothyroxine treatment was not monitored.

### Statistical analysis

All data are presented as mean ± SD. SPSS statistics 17.0 software was used for statistical analysis. For two separate groups, the Student's *t*-test (two-tailed, unpaired) was performed, and multiple groups were compared using analysis of variance. Correlation analysis was performed using a bivariate correlation analysis. A *P* value less than 0.05 was considered statistically significant.

## Results

### BAT glucose metabolism in obese and DM model mice

**Figure 1:**
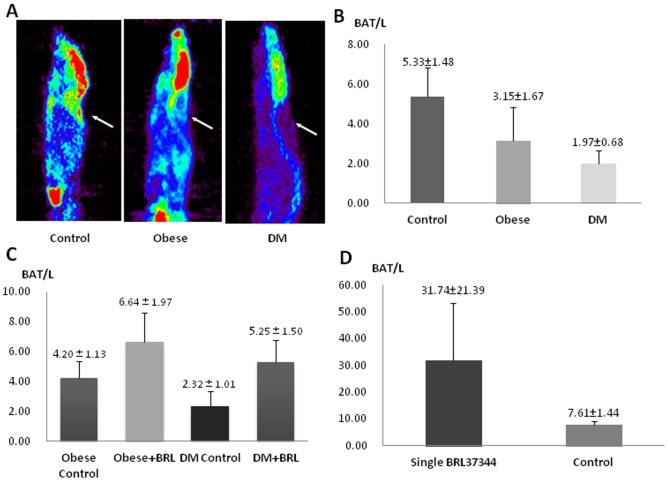
Comparison of brown adipose tissue (BAT) ^18^F-FDG uptake in the normal, obese, and diabetes mellitus (DM) mice and their response to BRL37344 intervention. A: Typical ^18^F-FDG microPET images of normal, obese, and DM mice after cold stimulation by steping on ice for 1 h. Arrows showed that the interscapular BAT uptake of ^18^F-FDG was decreased in the obese and DM mice. B: Semi-quantitative analysis of BAT-to-liver ratio (BAT/L) of ^18^F-FDG uptake showed that the BAT/L was significanly lower in the obese mice (*P* = 0.036) and further decreased in the DM mice (*P*<0.001). C: Two-week BRL37344 intervetion (three times per week) significantly increased the BAT/L in the obese mice (*P* = 0.010) and DM mice (*P* = 0.004) compared to the corresponding controls. D: Single-dose BRL37344 intervention also significantly increased the BAT/L in the normal mice (*P* = 0.002).

Baseline PET imaging showed no significant difference in the interscapular BAT/L uptake ratio between the groups of obese mice, DM mice, and control mice prior to any treatments. After induction of obesity and DM, the BAT uptake ratio (BAT/L) was successively decreased in the obese mice (3.15±1.67, *P* = 0.036) and DM mice (1.97±0.68, *P*<0.001) compared with the controls (5.33±1.48) (Fig.1A, 1B).

Two-week BRL37344 intervention significantly increased the BAT/L in both obese mice (6.64±1.97) and DM mice (5.25±1.50) compared with the corresponding controls without BRL37344 treatment (4.20±1.13 in obese control mice, *P* = 0.010; 2.32±1.01 in DM control mice, *P* = 0.004) (Fig. 1C). In the normal control mice, single-dose BRL37344 intervention also significantly increased BAT/L compared with the untreated controls (31.74±21.39 *versus* 7.61±1.44, *P* = 0.002) (Fig. 1D).

### BAT glucose metabolism after BRL intervention

#### *Ex vivo* biodistribution after single-dose BRL intervention

The 1 hour biodistribution of ^18^F-FDG in BAT and other tissues showed a similar pattern to that demonstrated by the microPET imaging. Single-dose BRL37344 significantly increased ^18^F-FDG uptake in the BAT in the normal mice (Table 2). There was no significant difference between the ^18^F-FDG uptake of the liver in two groups, indicating that ^18^F-FDG uptake in the liver was relatively stable, and the uptake ratio of BAT to liver (BAT/L) is a reliable parameter to evaluate the BAT function. The liver ^18^F-FDG uptake (nCi/cc) in different groups of mice did not show significant difference (*F* = 0.802, *P* = 0.558).

**Table 2:** The 1-h biodistribution of ^18^F-FDG in normal mouse with and without BRL37344 intervention.

	BAT	mWAT	Liver	Brain	Muscle
***BRL37344 group*** (n = 6, ×10^4^ CPM/g)	41.56±17.69	3.45±0.69	3.14±0.63	31.34±13.32	7.17±3.29
***Control group*** (n = 6, ×10^4^ CPM/g)	8.11±2.21	3.22±0.81	3.20±1.00	33.57±7.35	4.07±2.50
***P value***	0.001	0.600	0.910	0.730	0.100

BAT: brown adipose tissue; mWAT: mesenteric white adipose tissue; CPM: decay-corrected counts per minute.

#### BAT function and serum glucose level

After two weeks of BRL37344 intervention, both obese and DM mice showed a significantly lower level of fasting serum glucose levels. However, the serum TC and LDL-C levels were similar to those in the obese and DM controls (Table 3). After BRL37344 treatment, the fasting serum glucose levels in both obese and DM mice showed a significant negative correlation with the BAT/L ratios (r = −0.71, *P* = 0.003 and r = −0.74, *P* = 0.010, respectively). However, no correlation was found between the TC or LDL-C levels and BAT/L in either group.

**Table 3:** Comparison of serum levels of glucose, total cholesterol, and low-density lipoprotein-cholesterol in the control, obese, and DM mice.

	Glu(mmol/L)	*P*	TC(mmol/L)	*P*	LDL-C (mmol/L)	*P*
***Normal control***	4.90±2.01		3.29±1.09		0.16±0.06	
***Obesity control***	8.47±1.54	0.023	5.47±1.82	0.915	0.54±0.11	0.397
***Obesity + BRL37344***	6.88±0.57		5.55±0.85		0.49±0.13	
***DM control***	18.18±9.54	0.036	6.34±1.75	0.873	0.82±0.21	0.947
***DM + BRL37344***	8.84±2.86		6.51±1.60		0.82±0.11	

Glu: serum glucose level; TC: serum level of total cholesterol; LDL-C: low-density lipoprotein-cholesterol; DM: diabetes mellitus.

#### BAT function and body weight

**Figure 2:**
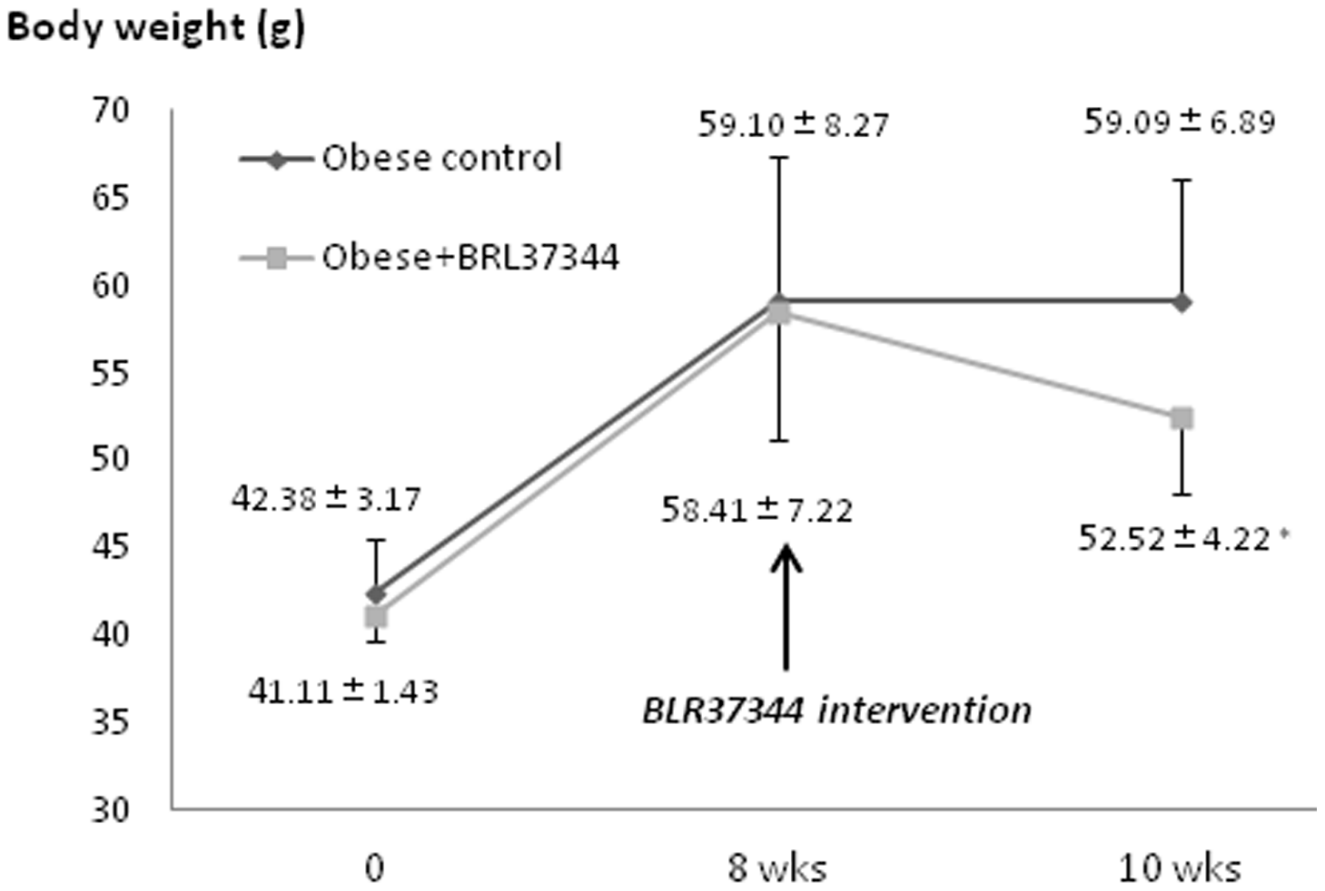
Comparison of the changes of body weight in the obese mice with and without BRL37344 treatment. Significant weight loss was observed after two weeks of BRL37344 treatment (* *P* = 0.001).

After eight weeks of a high-fat diet, the weight of the obese mice was significantly increased compared with the controls (42.52±6.16 g *versus* 58.78±7.53 g, *P* = 0.001). Two weeks of BRL37344 treatment significantly lowered the body weight of obese mice compared with untreated obese mice (52.52±4.22 g *versus* 59.09±6.89 g, *P* = 0.001) (Fig. 2).

### Levothyroxine intervention

**Figure 3:**
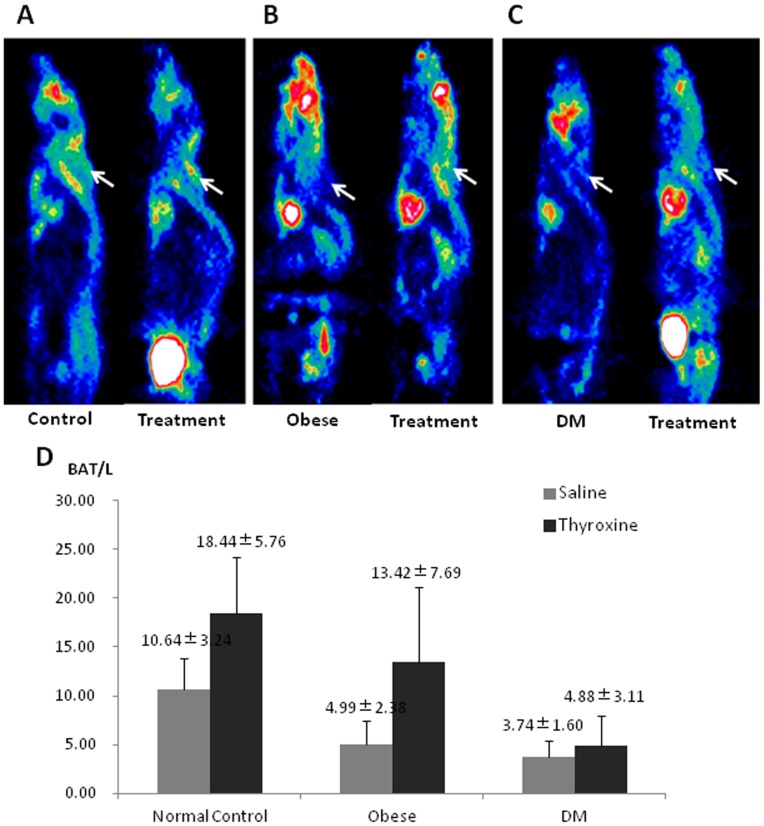
Comparison of brown adipose tissue (BAT) ^18^F-FDG uptake in the normal, obese, and diabetes mellitus (DM) mice underwent levothyroxine treatment. A, B, and C: Typical microPET images, with the arrows showing the interscapular BAT uptake of ^18^F-FDG in the mice. D: Semi-quantitative analysis of BAT-to-liver uptake ratio (BAT/L) showed that after two-week levothyroxine treatment, the BAT/L in the normal and obese mice were significantly increased compared to the corresponding controls (*P* = 0.025 and 0.013, respectively), whereas the increase of BAT/L in the DM mice was not significant (*P* = 0.450).

In the levothyroxine treatment group, two weeks of levothyroxine intervention significantly increased BAT/L in both normal mice (Fig. 3A) (18.44±5.76 versus 10.64±3.24, *P* = 0.025) and obese mice (Fig. 3B) (13.42±7.69 *versus* 4.99±2.38, *P* = 0.013), but not significant in the DM mice (Fig. 3C) (4.88±3.11 *versus* 3.74±1.60, *P* = 0.450). No significant change of blood glucose level was found between any of the three groups and their corresponding controls.

## Discussion

The heat production and energy consuming function of BAT opens a potential new window for treatment of obesity and DM. Although several related pre-clinical and clinical studies have been performed and reported [8], [14], [15], the role of BAT in obesity and DM remains to be clarified. In this study, we examined BAT glucose metabolism in obese and DM model mice using ^18^F-FDG microPET, and evaluated the dynamic metabolic changes of BAT during disease progression as well as interventions including β_3_-adrenergic and thyroid hormonal treatment.

The gradual deterioration of BAT function in the development of obesity and type 2 DM have been reported [10], [20], but the exact mechanism is still unclear and needs to be explored. In the present study, we found that the obese and DM model mice showed successively worse BAT function in terms of glucose metabolism. This might be due to the differences in elevated blood glucose levels for the obese and DM model mice (Table 3), and a higher level of blood glucose may have a greater inhibitory effect on the BAT glucose metabolism [21]. On this basis, long-term high blood-glucose level may down-regulate related gene transcription, including glucose transporter type 4 (GLUT4) and UCP1, which has been found to be decreased in obese and diabetic animal models [22]–. Meanwhile, thicker subcutaneous fat might also keep the body core insulated from the external cold environment, thus preventing the cold stimulation to BAT [15]. Moreover, the sympathetic nerve dysfunction seen in DM may also further block the activation of BAT from the adrenergic pathway.

β_3_-adrenergic receptor controls the process of BAT thermogenesis in rodents and also in humans [25]. The activation of β_3_-adrenergic receptor stimulates specifically the expression of a mitochondrial molecule, uncoupling protein-1 (UCP1), which is involved in cold-induced nonshivering thermogenesis in BAT. Therefore, β_3_-adrenergic receptor agonist is an option of interest for possible clinical translation to treat obesity and DM. Although BRL3734 has some binding affinity to β_1_ (Ki value 1750 nM) and β_2_-adrenergic receptors (Ki value 1120 nM), it is more commonly used as a specific β_3_-adrenergic receptor agonist (Ki value287 nM) in previous studies [26], [27] Some pre-clinical studies have shown that β_3_-adrenergic receptor agonists were effective in lowering blood glucose levels in obese and/or diabetic rodent models [28], [29]. However, they did not monitor the BAT function during the interventions.

In our study, we observed a significant inverse relationship between the BAT ^18^F-FDG uptake and blood glucose levels in both obese and DM mice under β_3_ receptor agonists. These findings not only indicate that the “dysfunctional” BAT can be re-activated, but also support the hypothesis that BAT activation has potential therapeutic value in obesity and DM. The sensitivity of BAT to BRL37344 stimulation in obese and DM mice was much lower than that in normal mice, probably owing to a blunted sympathetic nervous response to pharmaceutical and environment stimulus under conditions of obesity and DM, which was also indicated in other studies [30]–[33]. Since BRL37344 may still affect muscular metabolism by its low affinity to β_1_ and β_2_-adrenergic receptors, the skeletal muscles might also be activated under BRL37344 intervention. However, based on our e*x vivo* biodistribution analysis, BRL37344 intervention showed no significant effect on muscular ^18^F-FDG uptake. BRL37344 only increased BAT ^18^F-FDG uptake, but had no effect on muscle, brain, liver and WAT uptake. Therefore, the effect of BRL37344 on muscular metabolism is not the main factor for increased energy consumption and weight loss.

Thyroid hormonal activation is another recognized pathway for BAT thermogenesis [34]. Recently, several articles have reported the effect of thyroid hormone in treating obesity [35] and the relationship between thyroid hormone level and BAT function [36]. In this study, we found that two-week levothyroxine treatment could significantly increase the BAT glucose metabolism in the normal and obese mice, but not in the DM model mice. It might therefore be assumed that BAT in the DM mice is less responsive to levothyroxine intervention than that in the normal and obese mice. This phenomenon may be due to decreased function of type 2 iodothyronine deiodinase in DM mice, which has been found to be associated with increased insulin resistance and decreased BAT thermogenesis in DM [37], [38]. Moreover, although the BAT function in the normal and obese mice was increased after levothyroxine treatment in our study, we did not find significant changes of blood glucose levels between any of the levothyroxine treatment groups and their corresponding control groups. This finding may be explained as either an inadequate dose of levothyroxine or a weaker mechanism of the thyroid hormonal activation, which needs further investigations.

Considering that tail vessels may be deteriorated by the diabetes and DM modeling and multiple injections, we selected i.p. injection as the mode of delivery for greater consistency and repeatability in experimentation. Based on published literature and our prior study (see Fig. S1), the difference between the two injection routes on BAT ^18^F-FDG uptake was not significant at 1 hour after tracer administration [39].

There are some limitations in this study that we considered. First, the lack of CT coregistration decreased the accuracy of PET imaging for locating interscapular BAT in mice, especially in DM and obese mice whose BAT ^18^F-FDG uptake is not easily detectable. In this presented study, we used liver as a background tissue for calculating BAT uptake ratio, which was not an optimal method. However, considering that liver uptake is relatively more stable than other tissues in the body, and can represent the total ^18^F-FDG dose injected, we chose liver as a stable background tissue for BAT uptake comparison. This method has also been reported previously [40].In addition, our study did not perform *ex-vivo* analysis of BAT thermogenesis or glucose uptake by the means of classical histological or molecular approaches, which would provide more information for qualitative comparison in our imaging results. With additional *ex-vivo* confirmation such as BAT UCP1 expression in different animal models and under various interventions, the PET imaging results could be related to the pathophysiology change of BAT function and thus be more convictive. In the future, more efforts are needed to explore the mechanisms linking the BAT dysfunction with the sympathetic nerve control and hormonal activation pathways, as well as the effect of treatment-induced endogenous blood glucose changes on BAT metabolism.

## Conclusions

BAT function progressively deteriorates in the obese and DM model mice, but can be re-activated by the β_3_-adrenergic receptor agonist BRL and partly by the thyroid hormone levothyroxine. According to our findings, an effective activation of BAT function might lead to weight loss and blood glucose level lowering, and the ^18^F-FDG uptake in BAT is negatively correlated with blood glucose level in both obese and DM mice models, although more proof is needed to elucidate the whole mechanism during the process. Therefore, BAT dysfunction seems to be an important mechanism of obesity and DM, and re-activation of BAT function can be a potential strategy for the treatment of obesity and DM.

## Supporting Information

Figure S1 Comparison of BAT ^18^F-FDG uptake between i.v. injection and i.p. injection. The BAT ^18^F-FDG uptake was comparable at 30–120 minutes after injection between the two methods, and no significant difference was found at 60 minutes after the tracer injection (*P* = 0.63, n = 6 in each group).
(TIF)
