# Supplementary figures and images for: Activating Brown Adipose Tissue for Weight Loss and Lowering of Blood Glucose Levels: A MicroPET Study Using Obese and Diabetic Model Mice
Source: PLoS One. 2014 Dec 2;9(12):e113742. doi: 10.1371/journal.pone.0113742 (PMC4252055; doi:10.1371/journal.pone.0113742)

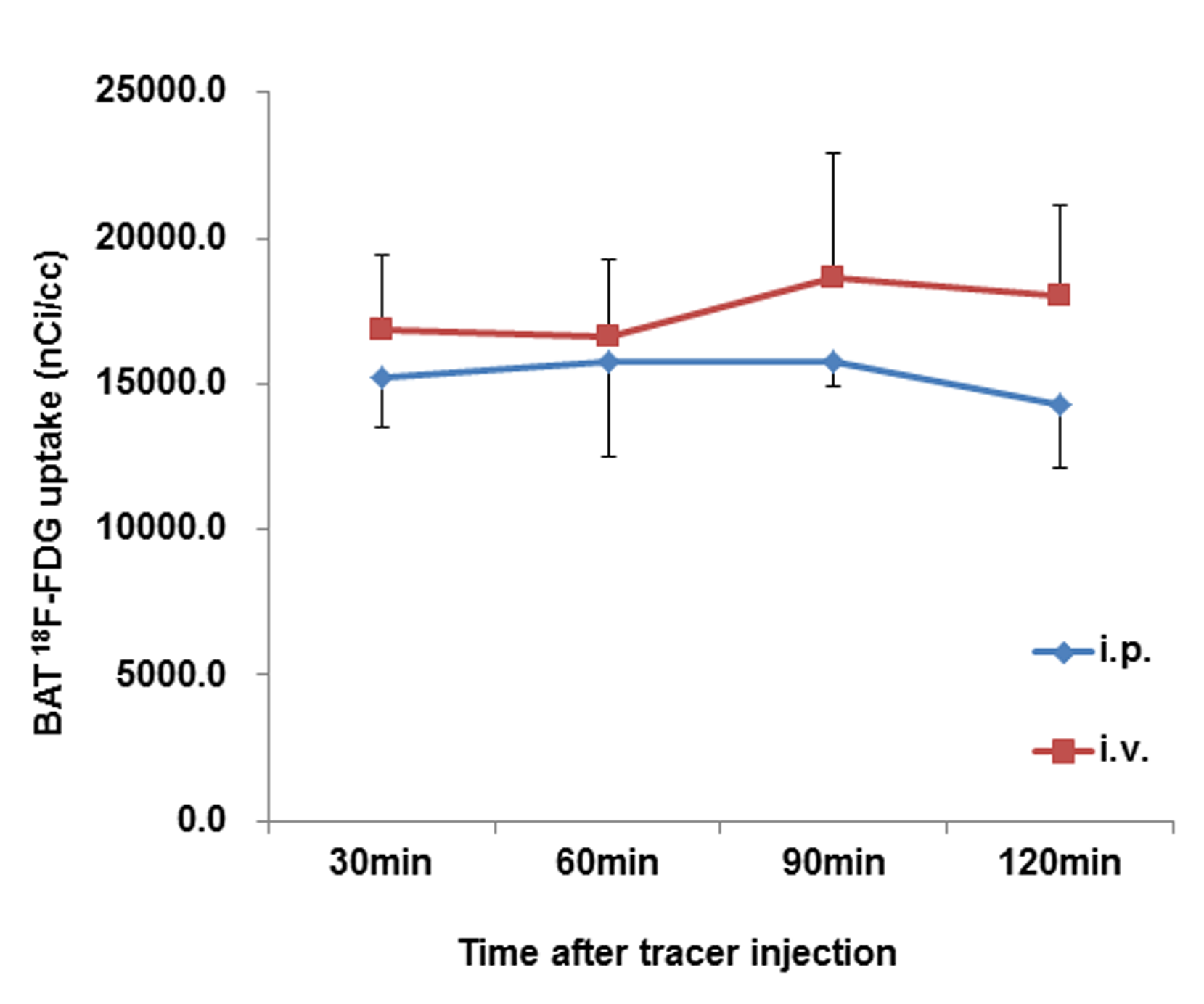

Supplement: Figure S1 — Comparison of BAT 18F-FDG uptake between i.v. injection and i.p. injection. The BAT 18F-FDG uptake was comparable at 30–120 minutes after injection between the two methods, and no significant difference was found at 60 minutes after the tracer injection (P = 0.63, n = 6 in each group). (TIF) [file pone.0113742.s001.tif]
